# Ultra-Broadband and Highly Efficient Beam Splitter Based on Quasi-Continuous Metasurface in the Near-Infrared Region

**DOI:** 10.3390/ma15186239

**Published:** 2022-09-08

**Authors:** Yan Liu, Tiesheng Wu, Yiping Wang, Zhihui Liu, Weiping Cao, Dan Yang, Zuning Yang, Rui Liu, Xu Zhong, Junyi Wang

**Affiliations:** 1 Guangxi Key Laboratory of Wireless Broadband Communication and Signal Processing, School of Information and Communication, Guilin University of Electronic Technology, Guilin 541004, China; 2 Key Laboratory of Optoelectronic Devices and Systems of Ministry of Education and Guangdong Province, College of Optoelectronic Engineering, Shenzhen University, Shenzhen 518060, China; 3 Guangdong and Hong Kong Joint Research Centre for Optical Fibre Sensors, College of Optoelectronic Engineering, Shenzhen University, Shenzhen 518060, China

**Keywords:** beam splitter, quasi-continuous metasurface, near-infrared region

## Abstract

Beam splitters are vital components in several optical systems. It is highly desirable, and compact beam splitters with ultra-broadband performances, high efficiencies, and large split angles are still being sought. In this paper, we demonstrate and numerically investigate an ultra-broadband and highly efficient optical beam splitter based on a quasi-continuous metasurface. The proposed design is constructed of quasi-continuous triangle-shaped gallium phosphide nanoantennas on a silica substrate. The simple structure can achieve a conversion efficiency and an anomalous transmission intensity above 90% and 0.8 covering the wavelength range of 1537–1826 nm, respectively. The maximum beam split angle in the operating bandwidth reaches 131.84° at the wavelength of 1826 nm. Particularly, the operating bandwidth is still as high as 125 nm with the anomalous transmission intensity above 0.92 and the conversion efficiency exceeding 99%. Moreover, the results show that the performance of the metasurface-based optical beam splitter can be further enhanced by optimizing structural parameters. We also demonstrate the adjustability of the beam splitter by adding refractive index (RI) materials on the surface of the device. The results show that the incident plane wave can be divided into three beams with intensity adjustability. The presented metasurface is very promising in the fields of multiplexers, interferometers, and optical communications, owing to its advantages of ultra-broadband, highly efficient, and large split angle simultaneously.

## 1. Introduction

Optical beam splitters, which divide a beam into two or more beams with different transmission directions, are essential components in many optical systems. It is widely used in optical communication, interferometer, multiplexing, and spectroscopy. It can also separate light with different states of polarization, wavelengths, and powers. Conventional beam splitters can be implemented in various methods and designs, such as photonic crystals [1,2], waveguide coupling structures [3,4,5,6], gratings [7,8,9], and wave plates [10]. Generally, such conventional beam splitters are relatively large, and the quality is relatively heavy, limiting their applications in the current trend towards photonics integration and miniaturization. Recent metasurface-based beam splitters can solve these problems by designing properly nanostructured components that allow us to achieve specific wavefront manipulation. Metasurfaces are used as a class of artificial materials with thickness less than wavelengths, which can perform flexible and effective manipulation of electromagnetic wave characteristics such as polarization [11,12,13], amplitude [14,15], phase [16,17,18], polarization mode [19,20], and propagation modes [21,22]. Due to these excellent features, metasurfaces have achieved rapid development for various applications, for example, polarization control [23,24,25], optical holograms [26], focusing lenses [27,28], biochemical testing [29], and orbital angular momentum [30]. According to the structural materials, metasurfaces are divided into plasmonic and dielectric metasurfaces. Plasmonic metasurface has a high optical loss due to its inherent dissipation, limiting transmission efficiency. In contrast, the dielectric metasurface easily achieves high transmission efficiency and easily manipulates scattering. Obviously, it is more appropriate to use the dielectric metasurfaces designing optical beam splitters.

According to the literature, there are two types of metasurface-based beam splitters. The first type of beam splitter is based on a discrete metasurface, usually consisting of two antenna arrays to realize opposite phase gradients or a periodic array based on binary elements with π phase difference between adjacent cells [31]. However, the discrete metasurface is limited by the aspect ratio of the design structure and is difficult to prepare accurately. Meanwhile, it is difficult to obtain a π phase difference for a wide band range based on two antenna arrays. Therefore, this type of discrete structure is inappropriate for designing metasurface-based beam splitters with high conversion efficiency and large bandwidth. The second type of optical beam splitter is based on a quasi-continuous structure that is constructed of two antenna arrays with opposite phase gradients, and each of the arrays can achieve complete 2π phase control. This type of metasurface-based beam splitter can achieve a high conversion efficiency, a wide bandwidth, and a large beam splitting angle simultaneously [32,33]. For example, Li et al. [32] proposed a beam splitter based on an all-dielectric quasi-continuous metasurface. Particularly, the conversion efficiency of the proposed structure can reach 95% with a bandwidth of 58 nm, and the split angle can reach ±78.91° at the wavelength of 738 nm. Li et al. [33] designed and numerically investigated a beam splitter based on a quasi-continuous metasurface with a conversion efficiency of 90% for the 969–1054 nm wavelength range, and the maximum split angle can reach ±47.10° at the wavelength of 996 nm. The designs of the previous literature still cannot meet the requirements for the performance of beam splitters in real environmental applications. Therefore, it is necessary and desirable to propose a new beam splitter based on an all-dielectric quasi-continuous metasurface with large split angles, a high anomalous transmission intensity, a high conversion efficiency, and a large operating bandwidth.

This work numerically demonstrates an equal-power beam splitter based on an all-dielectric quasi-continuous metasurface. The beam splitter possesses the advantages of ultra-broadband, high anomalous transmission intensity, high conversion efficiency, large split angle in the near-infrared range, and simple structure. The proposed structure is constructed of gallium phosphide (GaP) nanoantennas arrays supported by a silica substrate. It achieves conversion efficiency and anomalous transmission intensity higher than 90% and 0.8, covering the wavelength range of 1537–1826 nm. The maximum beam split angle in the operating bandwidth reaches 131.84° at the wavelength of 1826 nm. Particularly, the conversion efficiency of the power splitter is maintained at greater than 99% for the wavelength range of 1566–1690 nm. The quasi-continuous metasurface offers better beam splitting performance than any previously reported beam splitters based on discrete and quasi-continuous metasurfaces. In addition, the beam splitting characteristics can be adjusted effectively by adding refractive index material on the surface of the metasurface. These merits render the proposed quasi-continuous metasurface a good candidate as a near-infrared equal-power beam splitter. 

## 2. Design and Simulation Methods

**Figure 1:**
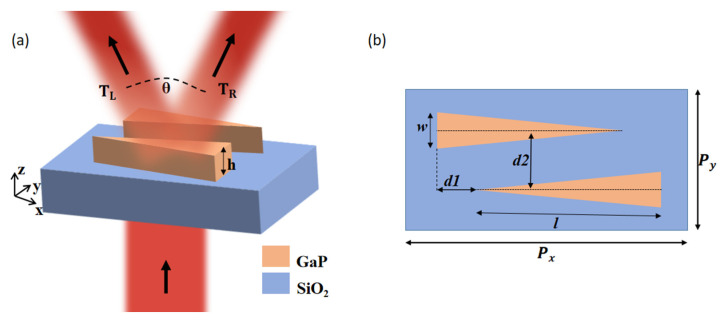
(**a**) Schematic of one unit cell of the proposed beam splitter and (**b**) top view of the beam splitter structure.

The schematic of the proposed design based on an all-dielectric quasi-continuous metasurface is shown in Figure 1a. In order to obtain equal-power beam splitting, two identical triangular dielectric array structures with opposite spatial variation are introduced on a quartz substrate for realizing opposite phase gradients. The two dielectric antenna arrays are constructed of gallium phosphide (GaP) with a large real part of the refractive index and a small loss coefficient. The top view of the proposed structure is illustrated in Figure 1b, with the periods of the proposed beam splitter along the x and y-directions set as P_x_ = 2000 nm and P_y_ = 710 nm. The quartz substrate has a thickness of 2000 nm. The length of the triangular dielectric antennas is l, with a value of 1300 nm; the width is w = 200 nm; and the height is 830 nm. The translational distances of the dielectric antennas in the x- and y-directions are d1 = 380 nm and d2 = 400 nm, respectively. In order to investigate the transmittance characteristics and the phase shift characteristics, we used commercial software Lumerical FDTD solutions to perform the finite difference time domain (FDTD) simulation. In the simulation, we set periodic boundary conditions in the x- and y-directions and a perfectly matched layer in the z-direction. GaP was used as the material for the metasurface, and the optical constants were taken from Ref. [34]. A plane wave with electric field component parallel to the x-axis is normally incident along the negative z-direction with the wavelength range of 1500–1850 nm.

## 3. Results and Discussion

### 3.1. Anomalous Transmission Properties of a Triangular Antenna Array

**Figure 2:**
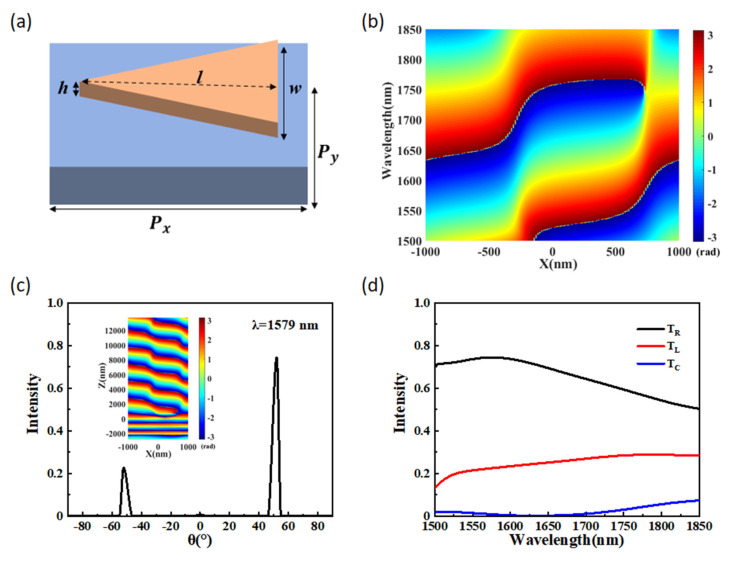
(**a**) Schematic of one unit cell of a simple metasurface with triangular GaP arrays on a quartz substrate. (**b**) The phase distribution along the x-direction of the unit cell within the 1500–1850 nm wavelength range. (**c**) Normalized far-field intensity distributions of the transmitted beams at a wavelength of 1579 nm, where the inset shows the phase distribution in the x-z plane. (**d**) Intensity of the anomalous transmission at 0, 1, and −1 diffraction orders.

For an equal-power beam splitter based on metasurface, the anomalous transmission properties greatly influence the splitting performance. As shown in Figure 2a, we first calculate the anomalous transmission properties of a simple metasurface structure that is constructed of triangular GaP arrays based on a quartz substrate. The structure parameters are set as described above, and the phase distribution of the transmitted optical beam along the x-axis is exhibited in Figure 2b. Obviously, one unit cell of the simple structure can realize phase shift for the 1500–1850 nm wavelength range. The phase shift can be explained by the propagation phase theory [35], the relationship between the propagation phase and the effective refractive index is $\varphi =h*n_{eff}*2\pi /\lambda$, where *n**_eff_*** is the effective refractive index of the fundamental mode obtained by an eigenmode analysis and λ is the wavelength in a vacuum. According to this constant equation, we can obtain the desired phase shift by selecting the material and by adjusting the structural shape and parameters. To obtain a deeper insight into the anomalous transmission properties in the metasurface structure, we calculated the normalized far-field intensity distributions of the transmitted beams and phase distribution in the x–z plane. As shown in Figure 2c, the wavelength is 1579 nm, and we can see that the transmitted light is divided into three beams. For simplicity, the anomalous transmission beam on the left side is described as T_L_, the other anomalous transmission beam on the right side is described as T_R_, and the beam with normal direction is described as T_C_. The left and right sides correspond to the negative and positive directions of the x-axis, respectively. At the operating wavelength of 1579 nm, the anomalous refraction angles reach ±49°, and the normalized anomalous transmission intensity on the left and right sides are 0.226 and 0.774, respectively. The inset of Figure 2c demonstrates the phase distribution of the triangular antenna arrays in the x–z plane at the same wavelength. The figure shows that the transmitted plane wave has arisen significant anomalous refraction and that the phase changes are relatively smooth in both the x- and z-directions. As shown in Figure 2d, the differences among the three transmission curves of T_L_, T_C_, and T_R_ are fairly discernible. In the range of 1500–1850 nm, the normalized intensity of T_C_ is in the range of 0.001–0.073. This result shows that most incident light is converted to anomalous transmission light when it passes through the simple periodicity structure.

In theory, the generalized Snell law can explain the anomalous transmission effect based on metasurface. According to the law, the transmission characteristics result from the phase distribution along the horizontal direction at the interface between two materials. The generalized Snell law can be demonstrated as follows [36]:

(1) $$n_{r}sin\theta_{r}-n_{i}sin\theta_{i}=\frac{\lambda_{0}}{2\pi }\frac{d\varphi }{dx}$$

where *θ_r_* is the refraction angle of the transmitted light, *θ_i_* is the incident angle of the incident light, *n_r_* and *n_i_* refer to the refractive indices of the surrounding medium and device material, *λ*_0_ is the wavelength transmitted in the vacuum, and *dφ/dx* represents the phase gradient of the metasurface. The incident light is incident and vertical to the interface, with the equation as follows:

(2) $$sin\theta_{r}=\frac{1}{n_{r}}\frac{\lambda_{0}}{2\pi }\frac{d\varphi }{dx}=\frac{\lambda_{0}}{P_{x}}\frac{1}{n_{r}}$$

where *P_x_* represents the period of the dielectric antenna arrays. For the proposed beam splitter based on a quasi-continuous metasurface, the split angle can be described by the grating equation, which modified the generalized Snell’s law as

(3) $$sin\theta_{r}=m\frac{\lambda_{0}}{P_{x}}+\frac{\lambda_{0}}{P_{x}}=\left(m+1\right)\frac{\lambda_{0}}{P_{x}}$$

where *m* represents the grating diffraction order. According to Equations (2) and (3), the split angle is decided by the wavelength of the incident light, the period of the dielectric antenna arrays, and the refractive index of the surrounding medium. When the refractive index of the surrounding medium *n_r_* = 1 and *λ/P_x_* is greater than 0.5, the value of the grating diffraction order is 0. In this case, the diffraction orders could only be 0, 1, and −1. We compare the simulation results and theoretical calculation results in the following discussion. 

### 3.2. Optical Properties of the Proposed Beam Splitter Based on Quasi-Continuous Metasurface 

**Figure 3:**
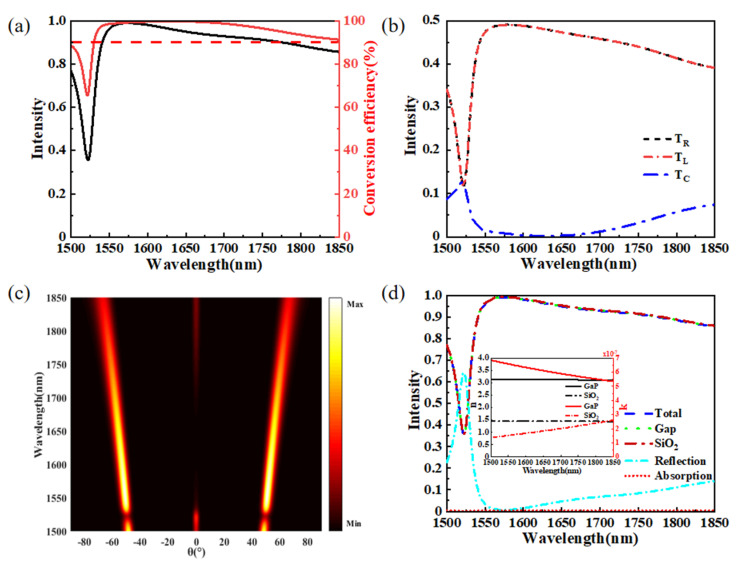
(**a**) Total transmission intensity and conversion efficiency. (**b**) The intensities of T_L_, T_R_, and T_C_ in the range of 1500–1850 nm. (**c**) The relationship between the intensity and transmission angle of the diffraction orders in the 1500–1850 nm wavelength range. (**d**) The transmission of each layer and total transmission, reflection, and absorption intensity.

As shown in Figure 1a, a triangular antenna array with opposite spatial variation is added to the quasi-continuous metasurface to constitute the structure of the proposed equal-power beam splitter. Combining two identical triangular dielectric antenna arrays with an opposite spatial variation can solve the problem that the intensities of anomalous transmission at the left and right sides are not equal, resulting in equal-power beam splitting. Our design goal is to simultaneously achieve a beam splitter with high anomalous transmission intensity, large split angle, and operational bandwidth. The anomalous transmission intensity is defined as t = T_L_ + T_R_. The conversion efficiency for incident light converts to anomalous transmission light, which is defined as η = t/(T_L_ + T_R_ + T_C_) ∗ 100%. The total transmission intensity and conversion efficiency of the beam splitter within the wavelength range from 1500 nm to 1850 nm is shown in Figure 3a. We can see that the total transmission intensity exceeds 0.8 for the 1536–1850 nm range. Interestingly, the conversion efficiency is maintained above 99%, with a bandwidth of 125 nm. In order to better observe the transmission characteristics of the proposed design, the intensities of T_L_, T_C_, and T_R_ are shown in Figure 3b. It is clear that the intensities of T_L_ and T_R_ are always equal over the entire range of 1500–1850 nm. This result shows that the quasi-continuous metasurface can realize equal-power beam splitting. At the wavelength of 1522 nm, it is clear that T_L_ = T_R_ = 0.11, so the anomalous transmission intensity has a minimum value of 0.22, while the intensity of normal transmission reaches a maximum value of 0.12. The bandwidth exceeds 255 nm for the intensity of normal transmission below 0.05. The bandwidth is still up to 171 nm when the intensity of normal transmission is lower than 0.01. The intensities of T_L_, T_C_, and T_R_ at any split angles are shown in Figure 3c. It is obvious that the split angle increases with increasing operating wavelength. When the structural parameters are unchanged, there is a one-to-one correspondence between the split angle and the operating wavelength. The total transmission, transmission of each layer, reflection, and absorption are plotted in Figure 3d. Two monitors were set in the middle of the quartz substrate and GaP nanoantennas to record the transmission intensity of every layer and make a judgment. The total transmission and transmission of each layer almost entirely coincided, which indicates that the proposed structure does not cause absorption loss and is depicted in Figure 3d with a red dotted line. This result is mainly attributed to the imaginary parts of the refractive indices of GaP and quartz, which can be negligible, as plotted in the insert of Figure 3d. Obviously, the reflection almost entirely happens at the quartz–air interface.

**Figure 4:**
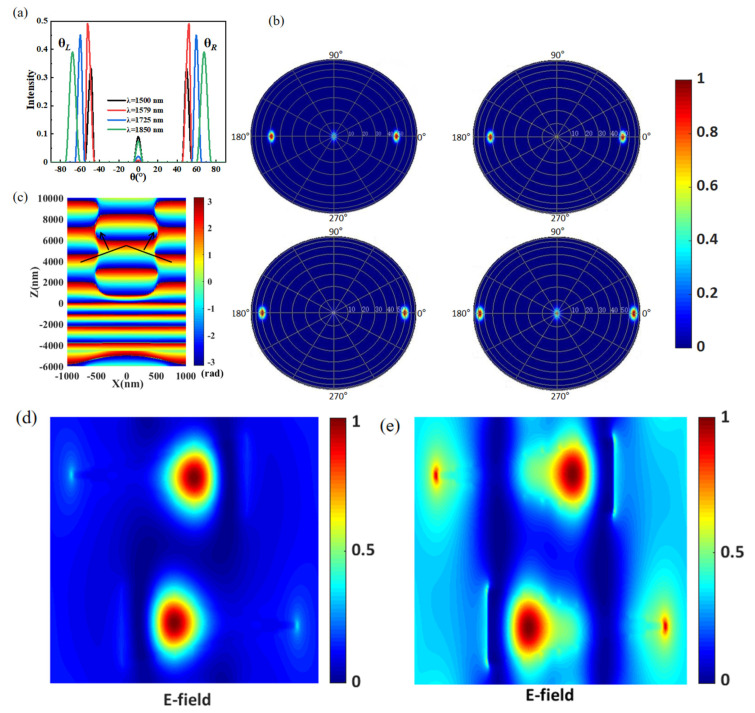
(**a**) Far-field transmitting intensities for different anomalous refraction angles and (**b**) the angular distributions of transmitted light at 1500, 1579, 1725, and 1850 nm wavelengths. (**c**) Phase distribution in the x-z plane at the wavelength of 1579 nm. (**d**) Electric field distribution at the wavelength of 1500 nm. (**e**) Electric field distribution at the wavelength of 1579 nm.

In order to verify the accuracy of the simulation results, we have compared the calculation and simulation results. The normalized far-field intensity and angle distribution of transmitted light at wavelengths 1573, 1579, 1725, and 1850 nm are shown in Figure 4a,b. Apparently, there are only three diffraction orders in the transmitted light of this structure: −1, 0, and +1. According to the generalized Snell law theory, this case requires λ/P*_x_* greater than 0.5. The calculated λ/P*_x_* for the four wavelength points are 0.75, 0.7895, 0.8625, and 0.925, respectively. The absolute values of anomalous refractive angles θ_L_ and θ_R_ are always the same. Therefore, we only use the absolute value to illustrate the refraction angle in detail. The simulated anomalous refractive angles for the four wavelength points are 48.5904°, 52.1388°, 59.5985°, and 67.6684°, respectively. This shows that the operating wavelength spans from 1500 nm to 1850 nm, and the split angle gradually could increase from 97.18° to 135.34°. According to Equation (3), the calculated anomalous refractive angles for four wavelength points are 48.59037°, 52.1388°, 52.1388°, and 67.66835°, respectively. The simulation angles are consistent with the calculation angles. The results show that the performance of the beam splitter obtained by the simulation is credible. The anomalous transmission characteristics of the metasurface-based beam splitter are closely related to its phase distribution. Figure 4c shows the phase distribution of the transmitted light at a wavelength of 1579 nm. At this wavelength, the intensity of normal refraction light can be ignored. Incident light dispels into two beams of anomalous transmission light with different propagating directions because the phase distribution is no longer planar and uniform, and the wavefront of the transmitted light is the same on the left and right sides and along the z-axis symmetric. The electric distributions at the wavelengths of 1500 nm and 1579 nm are shown in Figure 4d,e. As shown in Figure 4d, there is almost no electric field distribution in the gap between the adjacent triangular antenna, indicating that the coupling effect is very weak. However, there is a strong electric dipole resonance inside the triangular antenna, which consumes most of the light energy, so the light intensity of all diffraction orders is relatively low. As shown in Figure 4e, we can see that the internal electric field strength of the two triangular antennas is relatively low, and the gap electric field is significantly enhanced, indicating that there is a strong near-field coupling effect between them. Therefore, the transmitted beam at the ±1 order has a much higher intensity than the 0 order.

**Figure 5:**
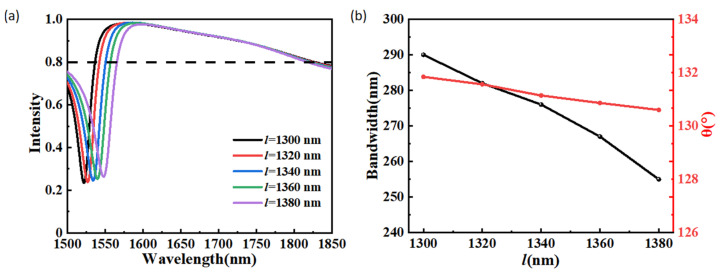
(**a**) Anomalous transmission intensity of the beam splitter at different values of *l*. (**b**) Operating bandwidth and the maximum beam split angle in the operating bandwidth at different values of *l*. Operating bandwidth is defined as the anomalous transmission intensity greater than 0.8.

In practical applications, the disturbance of structural parameters is often inevitable. We desire the proposed beam splitter to have high robustness and still have high beam split performance when the structural parameters are disturbed. Here, we have simulated and analyzed the effects of the structural parameters on the beam split performance for researching the robustness of the metasurface-based beam splitter. The calculated anomalous transmission intensity of the proposed structure at different lengths l is depicted in Figure 5a. The other structural parameters of the metasurface-based beam splitter remain unchanged. The black dashed line indicates that the anomalous transmission intensity equals 0.8. With the increase in *l*, the anomalous transmission spectra will be red-shifted. The maximum and minimum values of the anomalous transmission intensity change slightly for the 1500–1850 nm wavelength range. We found that when the anomalous transmission intensity is greater than 0.8, the conversion efficiency is always greater than 0.9. Here, we view the corresponding wavelength range as the operating bandwidth. As shown in Figure 5b, the influences of the length *l* on the operating bandwidth and the maximum beam split angle in the operating bandwidth are also investigated. The operating bandwidth and beam split angle decrease with the increase in the length *l*. When *l* varies from 1300 nm to 1380 nm, the operating bandwidth decreases from 290 nm to 255 nm, and the maximum beam split angle in the operating bandwidth decreases from 131.84° to 130.60°. Obviously, with *l* varies in dozens of nanometers, large operating bandwidth and big split angle are always obtained.

**Figure 6:**
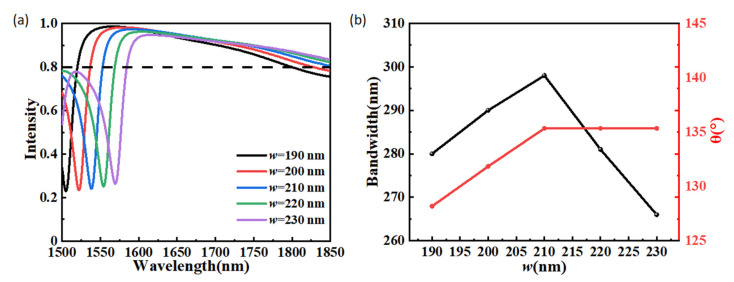
(**a**) The anomalous transmission intensity at different values of w within the wavelength range of 1500–1850 nm. (**b**) Operating bandwidth and the maximum beam split angle in the operating bandwidth as a function of the structure parameter *w*.

**Figure 7:**
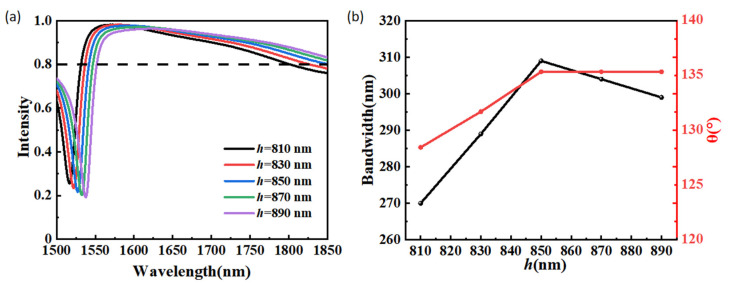
(**a**) The anomalous transmission intensity at different values of h at the wavelength range of 1500–1850 nm. (**b**) Operating bandwidth and the maximum beam split angle in the operating bandwidth as a function of the structure parameter *h*.

Figure 6a depicts the anomalous transmission intensity of the proposed structure with different antenna widths *w*. When w increases from 190 nm to 230 nm with a step of 10 nm, it is observed that the anomalous transmission spectra of the beam splitter is red-shifted within the wavelength range of 1500–1850 nm. It is observed that the maximum value of the anomalous transmission intensity slightly decreases from 0.986 to 0.947, and the corresponding wavelength shifts from 1581 to 1615 nm. The operating bandwidth and the maximum beam split angle in the operating bandwidth at different values of *w* are shown in Figure 6b. When dielectric the operating bandwidth and the maximum beam split angle in the operating bandwidth increase at first and then decrease, both of which reach the maximum values with a parameter of *w* = 210 nm. When the antenna width *w* varies from 190 nm to 230 nm, the variation range of the operating bandwidth is 266–280 nm, and the variation range of the maximum beam split angle in the operating bandwidth is 128.18°-135.34°. When *w* increases from 210 nm to 230 nm, the maximum beam split angle in the operating bandwidth is almost not affected by the changes of the structural parameter *w*. The effects of the antenna thickness h on the performance of the beam splitter are exhibited in Figure 7. antenna width *w* increases from 190 nm to 230 nm, As shown in Figure 7a, red-shift with increasing h is also observed from the anomalous transmission intensity spectra. When the antenna thickness *h* varies from 810 nm to 890 nm, the maximum value of the anomalous transmission intensity slightly decreases from 0.982 to 0.964. The operating bandwidth and the maximum beam split angle in the operating bandwidth as a function of the antenna thickness *h* are depicted in Figure 7b. When dielectric antenna thickness *h* varies from 810 nm to 890 nm, the trends are similar to those resulting from a variety of *w*. The operating bandwidth and the maximum beam split angle in the operating bandwidth also increase at first and then decrease, both of which reach the maximum values with a parameter of *h* = 850 nm. When the dielectric antenna thickness *h* varies from 810 nm to 890 nm, the variation range of the operating bandwidth is 270–299 nm, and the variation range of the maximum beam split angle in the operating bandwidth is 128.44–135.34°. When the dielectric antenna thickness *h* increases continuously from 850 nm to 890 nm, the maximum beam split angle in the operating bandwidth is almost not affected by the changes in the structural parameter *h*. 

**Figure 8:**
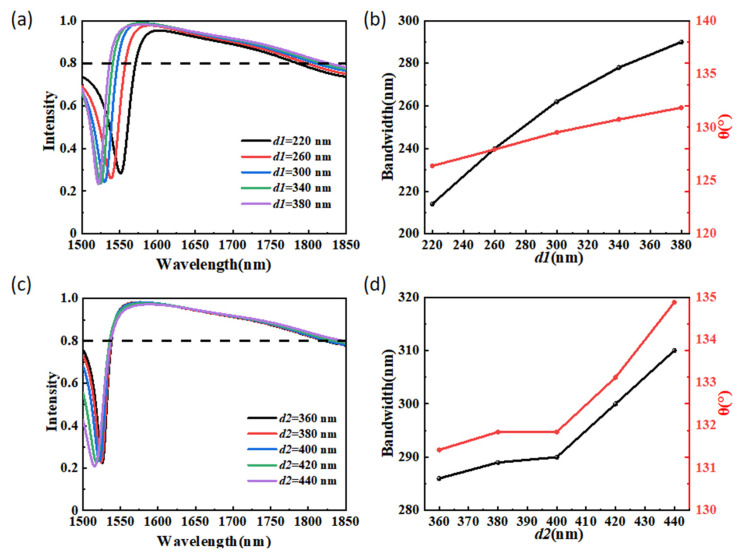
(**a**) The anomalous transmission intensity at different values of *d1* within the range from 1500 nm to 1850 nm. (**b**) Operating bandwidth and the maximum beam split angle in the operating bandwidth as a function of *d1*. (**c**) The anomalous transmission intensity at different values of *d2* within the range from 1500 nm to 1850 nm. (**d**) Operating bandwidth and the maximum beam split angle in the operating bandwidth as a function of *d2*.

Figure 8 illustrates the performances of the beam splitter with different translational distances for the dielectric antennas in the x- and y-directions. As shown in Figure 8a, when *d1* increases from 220 nm to 380 nm with a step of 40 nm, it is observed that the anomalous transmission spectra of the beam splitter is blue-shifted within the wavelength range of 1500–1850 nm. The maximum value of the anomalous transmission intensity increases first and then decreases. The influences of the translational distance of the dielectric antennas in the x-direction on the operating bandwidth and the maximum beam split angle in the operating bandwidth were also investigated and are shown in Figure 8b. The operating bandwidth and beam split angle increase with the increase in *d1*. When dl varies from 220 nm to 380 nm, the operating bandwidth increases from 214 nm to 290 nm and the maximum beam split angle in the operating bandwidth increases from 126.38° to 130.60°. The effects of the translational distance *d2* on the anomalous transmission intensity were also investigated. As shown in Figure 8c, we can see that there is almost no shift in the anomalous transmission spectra of the beam splitter within the wavelength range of 1538–1850 nm. In Figure 8d, we calculated the operating bandwidth and the maximum beam split angle in the operating bandwidth as a function of the parameter *d2*. As shown in Figure 8d, with *d2* increasing from 360 nm to 440 nm, the operating bandwidth increases from 286 nm to 310 nm, and the maximum beam split angle in the operating bandwidth increases from 131.42° to 134.88°. These results show that the metasurface-based beam splitter has high robustness.

In order to show the superior performances of the proposed design based on an all-dielectric quasi-continuous metasurface, the performances of the beam splitter, including operating wavelength, conversion efficiency, anomalous transmission intensity, the maximum beam split angle in the operating wavelength range, and operating bandwidth are compared with the results of the previous reports based on metasurface beam splitters, as shown in Table 1. In [16], the beam splitter consists of two discrete ring-column arrays with opposite phase gradients, and the design wavelength was in the range 1310–1850 nm. In [27], the beam splitter was composed of two discrete cylindrical array metasurfaces with opposite phase gradients in the wavelength range of 700–1200 nm. In [31], the beam splitter was realized by a metasurface composed of a periodic array of binary cells so that there was a π phase difference between adjacent cells to achieve beam splitting, and the beam splitters were designed over the range of 450–650 nm. Obviously, compared with the simulation results from the previous reports, the proposed structure shows huge advantages in many aspects, such as higher conversion efficiency, higher anomalous transmission intensity, and bigger beam split angle in a larger operating bandwidth.

**Figure 9:**
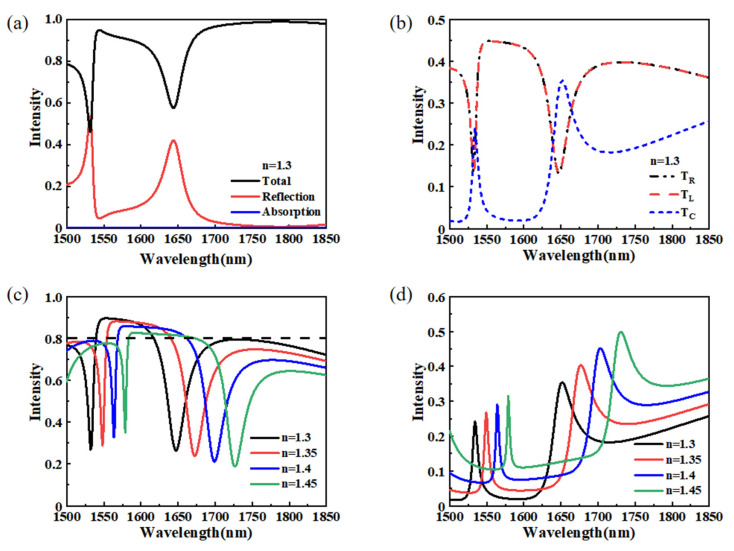
(**a**) Total transmission, reflection, and absorption spectra of the proposed metasurface with n = 1.3. (**b**) The intensities of T_L_, T_R_, and T_C_ for the range of 1500–1850 nm with n = 1.3. (**c**) The anomalous transmission intensity of the proposed structure with different refractive indices. (**d**) Reflection spectra of the proposed design with different refractive indices.

**Figure 10:**
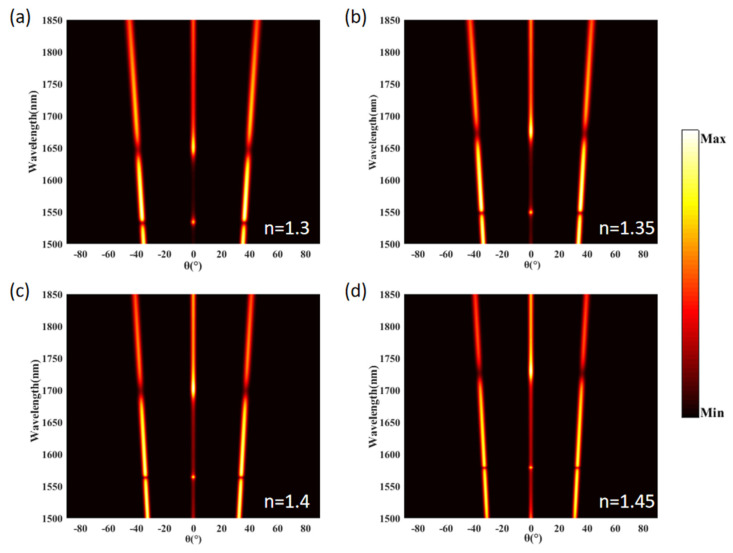
The relationship between the transmission intensity and refractive angle of the diffraction orders in the wavelength range of 1500–1850 nm with different refractive indices. (**a**) n = 1.30, (**b**) n = 1.35, (**c**) n = 1.40, and (**d**) n = 1.45.

According to Equation (1), the anomalous transmission effect is related to the surrounding medium’s refractive index (RI). Here, we investigated the influence of the refractive index of the surrounding medium on the transmission characteristics. Figure 9a shows the total transmission, reflection, and absorption spectra of the proposed structure with an RI of n = 1.30. The absorption can almost be ignored within the 1500–1850 nm range. There are two extreme points in the total transmission and reflection spectra at 1532 nm and 1644 nm, respectively. The intensities of T_L_, T_C_, and T_R_ at a refractive index of 1.3 are exhibited in Figure 9b. There is a significant difference in the intensities between the refractive indices n = 1.3 and n = 1 (shown in Figure 2d). The transmitted light becomes three beams of light with different propagating directions. There are two extreme points in the anomalous transmission spectra at the wavelengths 1532 nm and 1647 nm. The peak wavelengths of the reflection spectrum are 1534 nm and 1652 nm, respectively. Figure 9c,d depict the total anomalous transmission and reflection intensities of the proposed structure with different refractive indices. As the RI of the surrounding medium increases from 1.30 to 1.45, the anomalous transmission and reflection spectra exhibit a linear red-shift. As shown in Figure 9d, when the RI increases from 1.30 to 1.45, the peak wavelength on the left of the reflection spectrum shifts from 1543 nm to 1579 nm and the peak wavelength on the right of the reflection spectrum shifts from 1579 nm to 1731 nm. The shift of the peak wavelength on the right side reaches 79 nm. As we know, the refractive index sensitivity is defined as dn/dλ, and the calculated sensitivity for detecting the peak wavelength on the right side is 526.67 nmRIU^−1^. The results indicate that the proposed structure can be used as a refractive index sensor. The relationship between the intensity and refractive angle of the diffraction orders in the 1500–1850 nm wavelength range with different refractive indices is shown in Figure 10. It is clear that the variation in the refractive index has a small impact on the anomalous refraction angle. Still, it significantly affects the intensity ratio of the anomalous transmission beam and the normalized transmission beam. We can adjust the intensities of the anomalous and normalized transmitted light beams by putting the refractive index material on the surface of the proposed metasurface.

## 4. Conclusions

In summary, we presented and demonstrated an ultra-broadband, highly efficient, equal-power beam splitter based on an all-dielectric quasi-continuous metasurface in the near-infrared region. The proposed quasi-continuous metasurface was constructed of two identical triangular dielectric antenna arrays with opposite spatial variations for realizing opposite phase gradients. An anomalous transmission intensity over 0.8 and a conversion efficiency above 92.19% were obtained within the wavelength range of 1537–1826 nm. The maximum beam split angle in the operating bandwidth reached 131.84° at a wavelength of 1826 nm. The operating bandwidth still exceeded 125 nm, with the anomalous transmission intensity above 0.92 and the conversion efficiency exceeding 99%. The quasi-continuous metasurface performed better than any previously reported beam splitters based on discrete and quasi-continuous metasurfaces. In addition, by adding refractive index materials to the surface of the device, the incident light could be divided into three beams, and the intensity of each could be adjusted by changing the refractive index of the surrounding medium. These merits, including ultra-bandwidth, high anomalous transmission intensity, high conversion efficiency, large beam split angle, and simple structure, render the quasi-continuous metasurface a good candidate to be applied in many compact integrated devices.

## Figures and Tables

**Table 1:** Performances of the proposed structure and the previous reports based on discrete and quasi-continuous metasurfaces.

Splitter	Material	Wavelength (nm)	Conversion Efficiency (%)	Anomalous Transmission	Beam Split Angle (°)	Operating Bandwidth (nm)
He et al. [13]	Titanium Dioxide	480–600	-	0.6–0.88	58	120
Chen et al. [16]	silicon	1550	93.21	-	34.18	Narrow bandwidth
Ding et al. [21]	silicon	1309–1436	-	0.72–0.81	91.6	127
Liu et al. [25]	Aluminum antimonide	675–786	80–93.4	-	121.8	111
Zhang et al. [27]	lithium niobate	800	-	0.60	23.34	Narrow bandwidth
Ozer et al. [31]	Titanium Dioxide	532	92.8	0.90	93.6	Narrow bandwidth
Li et al. [32]	silicon	604–738	95–99.5	0.66–0.78	157.82	134
Li et al. [33]	silicon	969–1054	90–93.8	0.74–0.94	108.68	85
Our work	Gallium Phosphide	1537–1826	92.19–100	0.8–0.98	131.84	289

## Data Availability

Data sharing is not applicable to this article.
